# Impact of a Pharmacist-Led Antimicrobial Stewardship Program on the Number of Days of Antimicrobial Therapy for Uncomplicated Gram-Negative Bacteremia in a Community Hospital

**DOI:** 10.7759/cureus.14635

**Published:** 2021-04-22

**Authors:** Tetsuya Fukuda, Kentaro Tanuma, Satoru Iio, Jumpei Saito, Makoto Komura, Akimasa Yamatani

**Affiliations:** 1 Department of Pharmacy, National Center for Child Health and Development, Setagaya-ku, JPN; 2 Department of Pharmacy, National Hospital Organization Nishisaitama-Chuo National Hospital, Tokorozawa-shi, JPN

**Keywords:** adolescent, bacteremia, pharmacists, physicians, specialization, antimicrobial stewardship, community hospital, team medicine

## Abstract

Introduction

The need for pharmacist-led antimicrobial stewardship programs (ASP) is increasing.

Objective

We performed a retrospective study to assess whether pharmacist-led ASPs can decrease the duration of treatment for uncomplicated gram-negative bacteremia among patients admitted in a community hospital.

Methods

This research was conducted at a 325-bed regional general hospital in Japan, from January 2013 to June 2015. There are no infectious diseases specialists affiliated with the hospital. The outcomes of the pharmacist-led ASP group, who received pharmacist intervention, and the control group, who did not receive pharmacist intervention, were compared. The study included patients aged 18 years or older who were diagnosed with gram-negative bacteremia. The pharmacist performed an antimicrobial time-out at 72 hours after blood culture collection and optimized treatment based on the patient's clinical response and test results. The primary outcome was the duration of antibiotic treatment.

Results

In total, 34 patients in the pharmacist-led ASP group and 32 in the control group were included in the final analysis. The median number of days of antimicrobial treatment was 8 (interquartile range [IQR]: 7-14) days in the pharmacist-led ASP group and 14 (IQR: 10-15) days in the control group. The number of days of antimicrobial treatment significantly reduced in the pharmacist-led ASP group (p < 0.001). The de-escalation rates were 11 (32.4%) cases in the pharmacist-led ASP group and 4 (12.5%) cases in the control group. Hence, the trend was higher in the pharmacist-led ASP group than in the control group (p = 0.08).

Conclusion

The pharmacist-led ASP reduced the number of days of antimicrobial therapy for uncomplicated gram-negative bacteremia among patients admitted in a community hospital without an infectious diseases specialist.

## Introduction

The proportion of drug-resistant bacteria is increasing worldwide. This phenomenon is attributed to the inappropriate use of antimicrobial agents. Moreover, the development of new antimicrobial agents has been decreasing. Thus, drug-resistant bacteria have become an issue, and antimicrobial stewardship programs (ASP) [1] have been attracting attention.

A previous study has shown that pharmacists can lead ASPs [2]. In Japan, there are only a few infectious diseases specialists [3]. Therefore, the need for pharmacist-led ASP, which can improve the quality of medical care for *Staphylococcus aureus *bacteremia [4-5], is increasing. Moreover, a previous research has presented the health and economic benefits of pharmacist-led ASP among patients admitted in a community hospital in Japan [6]. However, only a few reports have shown that pharmacist-led ASPs reduced the number of antimicrobial days.

Hence, we aimed to perform a retrospective study to assess whether pharmacist-led ASPs can decrease the duration of treatment for uncomplicated gram-negative bacteremia among patients admitted in a community hospital without an infectious diseases specialist in Japan.

## Materials and methods

Study design and patient selection

This study was conducted at National Hospital Organization Nishisaitama-Chuo National Hospital, a 325-bed regional general hospital in Japan, from January 2013 to June 2015. There are no infectious diseases specialists affiliated with the hospital.

The outcomes of the pharmacist-led ASP group, which received pharmacist intervention, and the control group, who did not receive pharmacist intervention and were management to usual treatment, were compared.

The inclusion criterion included patients aged 18 years or older who were diagnosed with gram-negative bacteremia. Meanwhile, the exclusion criteria were immunocompromised patients; those with sepsis, intravascular device, abscesses, carbapenem-resistant Enterobacteriaceae, and carbapenemase-producing Enterobacteriaceae; and those taking antimicrobials for less than 5 days or more than 21 days for suspected non-infectious or complicated bacteremia.

Pharmacist-led ASP

The pharmacists performed an antimicrobial time-out at 72 hours after blood culture collection and optimized therapy based on the patient's clinical response and test results. Antimicrobial time-out is a technique in which the pharmacist discusses the efficacy, duration, de-escalation, and side effects of antimicrobial therapy on days 3, 5, 7, and 10 with the attending physician. Seven pharmacists (one infectious diseases [ID] pharmacist and six ward pharmacists) were included in the pharmacist-led ASP team.

Outcomes

The primary outcome was the duration of antibiotic treatment.

The secondary outcomes were de-escalation, clinical success and failure, recurrence, infectious diseases re-admission,* Clostridioides difficile* infection, and 30- and 60-day mortality rates.

Data collection

We collected data including those of age, sex, estimated creatinine clearance, medical history, Charlson Comorbidity Index [7], acquisition and source of bacteremia, and blood culture isolates. All data were collected from medical charts.

Statistical analysis

The sample size was calculated by assuming that the pharmacist-led ASP can reduce the mean treatment time (16 days, standard deviation: 2.23) for gram-negative bacteremia by 2 days (12.5%) in the pilot period (January 2013 to June 2013). To account for missing data, the total number of patients in each group was set at 35. Continuous data were analyzed using the Mann-Whitney U test, and categorical data were examined using the Fisher's exact test or the chi-square test. All tests were two-tailed, and a p-value of < 0.05 was considered statistically significant. All statistical analyses were performed using R version 4.0.3 (R Foundation for Statistical Computing, Vienna, Austria).

Ethical consideration

This study was approved by the Research Ethics Committee of National Hospital Organization Nishisaitama-Chuo National Hospital, Tokorozawa-shi (approval no.: 26-05).

## Results

**Figure 1:**
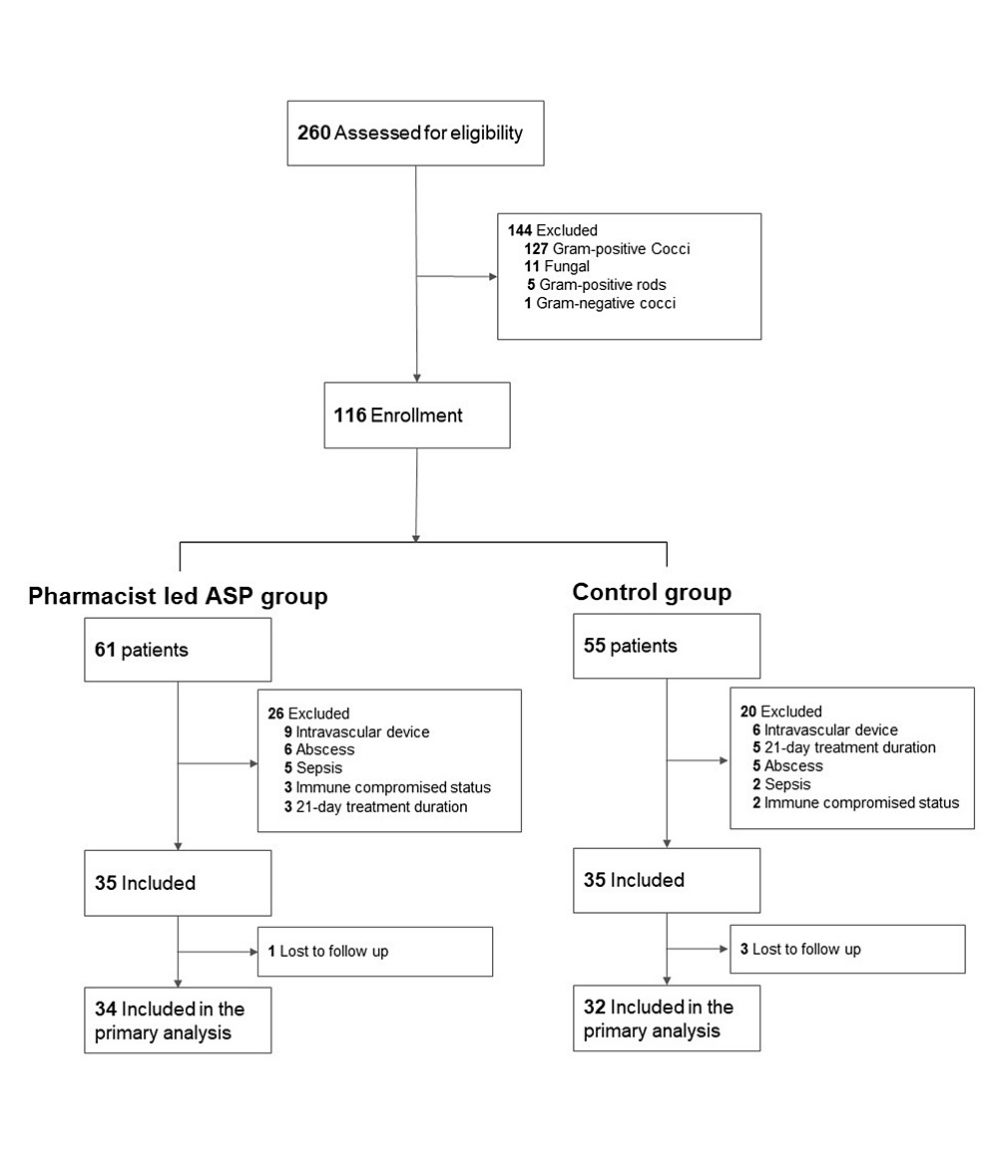
Flowchart of patient selection In total, 116 patients were assessed. Then, 34 and 32 patients were included in the pharmacist-led ASP and control groups, respectively. ASP: antimicrobial stewardship program

Figure 1 shows the flowchart of patient selection. In total, 116 patients were assessed. Then 34 and 32 patients were included in the pharmacist-led ASP and control groups, respectively. The background characteristics of the pharmacist-led ASP and control groups were similar (Table 1).

**Table 1:** Baseline characteristics of the participants The background characteristics of the pharmacist-led ASP and control groups were similar. ASP: antimicrobial stewardship program; IQR: interquartile range

	Pharmacist-led ASP group	Control group
	n = 34	n = 32
Male/female	20/14	18/14
Age (years), median (IQR)	82 (75–84)	79 (74–84)
Body weight (kg), median (IQR)	54 (49–66)	56 (52–63)
Estimated creatinine clearance (mL/min), median (IQR)	47 (38–60)	58 (36–72)
Primary diagnosis		
Gastroenterology	12	12
Surgery	7	6
Urology	7	7
Cardiology	4	4
Obstetrics and Gynecology	2	0
Others	2	3
Comorbidities		
Charlson Comorbidity Index, median (IQR)	1 (0–2)	1 (0–2)
Cancer	6	5
Diabetes mellitus	8	8
Heart failure	2	3
Liver cirrhosis	2	2
Bacteremia acquisition		
Community acquired	18	20
Nosocomial	15	12
Unknown	1	0
Source of bacteremia		
Urinary	26	22
Abdominal	4	5
Unknown	4	3

Table 2 shows the causative microorganisms. *Escherichia coli* was the most common causative agent in both groups, and the number of extended-spectrum beta-lactamase (ESBL) cases was similar.

**Table 2:** Data about the pathogenic microorganisms detected in multiple blood cultures *Escherichia coli* was the most common causative agent in both groups. ASP: antimicrobial stewardship program

	Pharmacist-led ASP group	Control group
	n = 34	n = 32
Blood culture isolate, no. (%)		
Escherichia coli	18 (52.9)	14 (43.8)
Extended-spectrum beta-lactamase	2 (5.9)	3 (9.3)
Klebsiella spp	2 (5.9)	1 (3.1)
Klebsiella pneumonia	1 (2.9)	3 (9.3)
Extended-spectrum beta-lactamase	0 (0)	1 (3.1)
Klebsiella oxytoca	3 (8.8)	2 (6.3)
Extended-spectrum beta-lactamase	1 (2.9)	0 (0)
Enterobacter spp	1 (2.9)	2 (6.3)
Enterobacter cloacae	2 (5.9)	0 (0)
Proteus spp	0 (0)	1 (3.1)
Acinetobacter baumannii	1 (2.9)	2 (6.3)
Acinetobacter lwoffii	0 (0)	1 (3.1)
Others	3 (8.8)	2 (6.3)

**Figure 2:**
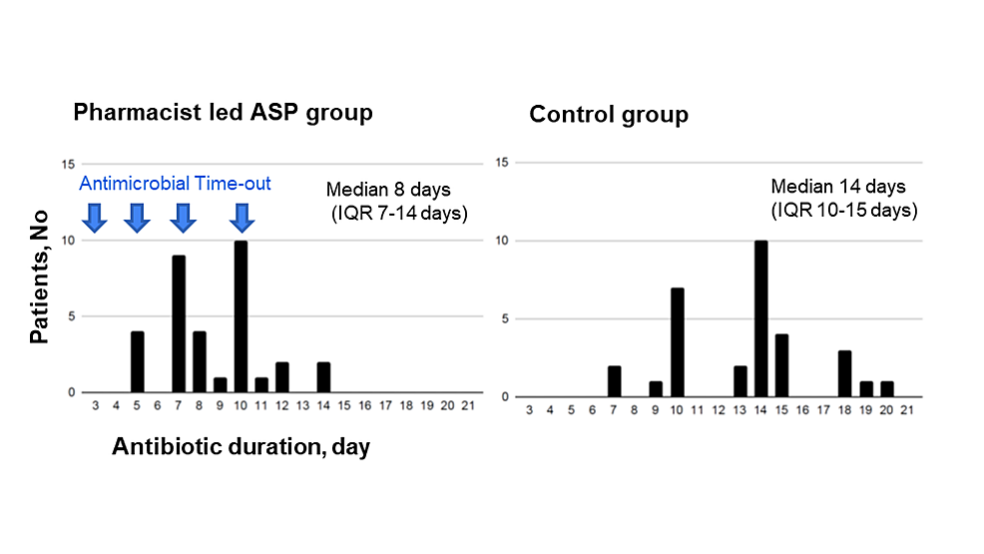
Distribution of the number of days of antimicrobial therapy The median number of days of antimicrobial administration was 8 (IQR: 7-14) days in the pharmacist-led ASP group and 14 (IQR: 10-15) days in the control group. ASP: antimicrobial stewardship program; IQR: interquartile range

Figure 2 shows the distribution of the number of days of antimicrobial therapy, and Table 3 depicts the median number of days of antimicrobial therapy and the secondary outcomes of each group. The median number of days of antimicrobial administration was 8 (interquartile range [IQR]: 7-14) days in the pharmacist-led ASP group and 14 (IQR: 10-15) days in the control group. The median number of sources of bacteremia significantly reduced in the pharmacist-led ASP group (p < 0.001). The de-escalation rates were 11 (32.4%) cases in the pharmacist-led ASP group and 4 (12.5%) cases in the control group. The pharmacist-led ASP group had higher rates than the control group (p = 0.08). However, the results did not significantly differ.

**Table 3:** Clinical outcomes Depicts the median number of days of antimicrobial therapy and the secondary outcomes of each group. ASP: antimicrobial stewardship program; IQR: interquartile range

	Pharmacist-led ASP group	Control group	P-value
	n = 34	n = 32	
Primal outcome			
Antibiotic treatment duration (days), median (IQR)	8 (7–10)	14 (10–15)	<0.01
Secondary outcomes, no. (%)			
De-escalation	11 (32.4)	4 (12.5)	0.08
Clinical success	32 (94.1)	30 (93.8)	1.0
Clinical failure	2 (5.9)	2 (6.3)	1.0
Recurrent bacteremia	0 (0)	0 (0)	1.0
Infectious diseases re-admission	0 (0)	0 (0)	1.0
*Clostridioides difficile * infection	1 (2.9)	0 (0)	1.0
30-Day mortality	1 (2.9)	1 (3.1)	1.0
60-Day mortality	2 (5.9)	2 (6.3)	1.0

## Discussion

This study had two important findings. First, the number of days of antimicrobial therapy for uncomplicated gram-negative bacteremia decreased in the pharmacist-led ASP group. Second, in a limited sample size, the group did not experience an increase in treatment failure, mortality, relapse, and re-admissions.

The pharmacist-led ASP was associated with a reduced number of days of antimicrobial therapy for uncomplicated gram-negative bacteremia. We assumed that it can reduce the number of days of antimicrobial therapy by 2 (12.5%) in the pharmacist-led ASP group. However, our results showed that the number of days decreased by 6. This may be due to the fact that the antimicrobial time-out by the pharmacists prompted the attending physician to reconfirm the general condition of the patient, leading to the decision to discontinue the antimicrobial therapy for patients in good condition.

Previously, von Dach et al. reported that in adult patients with uncomplicated gram-negative bacteremia, both individualized antimicrobial dosing based on C-reactive protein levels and 7-day fixed dosing were not inferior to 14-day fixed dosing in terms of 30-day clinical failure rate [8]. Although this was a randomized controlled trial including 504 patients from multiple centers, the 30-day clinical failure rate was low. However, its usefulness should be interpreted with caution. Therefore, antimicrobial time-out, which is performed by pharmacists, can reduce the risk of adverse drug reactions and can be used in clinical settings.

Further, in a limited sample size, the pharmacist-led ASP was not correlated with increased treatment failure, mortality, relapse, and re-admissions. Brotherton et al. reported that the pharmacist-led *S. aureus* bacteremia management bundles was associated with a lower mortality or 90-day readmission rate [9]. However, our study showed that the pharmacist-led ASP was not associated with a significant reduction in mortality rate because gram-negative rod (GNR) bacteria have a shorter duration of standard antimicrobial therapy compared with gram-positive cocci (GPC) bacteremia and a lower risk of abscess formation including endocarditis. Notably, the reduced duration of antimicrobial therapy may not have a negative effect on the prognosis or adverse effects. Moreover, an increasing trend in de-escalation rates was observed. Previous studies have reported that antimicrobial time-out is correlated with a reduced antimicrobial use and medical cost [10-12], and the combination of these interventions is more effective in reducing cases of resistance and beneficial in terms of costs.

The current study had several limitations. First, patients who were critically ill or had highly resistant strains were excluded. These patients required an individualized approach and thus the data was difficult to generalize. Second, this was a single-center study. We believed it was not statistically appropriate to perform a multivariate analysis based on the number of patients in this study. Third, the different primary diagnoses can be confounding factors. We may need to consider stratification as a response. However, a multicenter study should be conducted, and more cases must be included. In this manner, patient prognosis and safety can be better defined.

## Conclusions

The pharmacist-led ASP was associated with a reduced number of days of antimicrobial therapy for uncomplicated gram-negative bacteremia among patients admitted in a community hospital without an infectious diseases specialist. Hence, pharmacists must discuss not only the type of antimicrobial agents but also the duration of treatment with the attending physician.

However, interpretation is limited by the following factors: lack of inclusion of highly resistant cases, single-center study, low observed event rate, such as treatment failure, mortality, relapse, and re-admissions, and the variability in departments.
